# Direct Chemical Interference with the MTT viability assay: Identification and mitigation strategies in New Approach Methodologies (NAMs)

**DOI:** 10.1016/j.namjnl.2026.100148

**Published:** 2026-10-15

**Authors:** Courtney Nabua, Megan Creelman, Huang Huang, Nathan R. Wilt, Gertrude-Emilia Costin

**Affiliations:** Institute for In Vitro Sciences, Inc., Gaithersburg, MD, USA

**Keywords:** 3-(4,5-dimethyl-2- thiazolyl)-2,5-diphenyl-2H-tetrazolium bromide (MTT), MTT reduction, Viability assays, Interference, Direct MTT reducers

## Abstract

The reduction of the vital dye 3-(4,5-dimethyl-2- thiazolyl)-2,5-diphenyl-2H-tetrazolium bromide (MTT) is commonly used to assess the cellular viability and proliferation and thus to evaluate the cytotoxicity of discrete chemicals, complex mixtures, new drug candidates or finished cosmetic/personal care products. The MTT assay plays a central role in New Approach Methodologies (NAMs) due to its relatively simple design, short execution time and high throughput versatility. However, certain chemicals can directly interfere with the colorimetric MTT endpoint in the absence of a metabolically active biological system, potentially affecting data interpretation. In this manuscript, we identified and analyzed peer-reviewed manuscripts reporting chemical classes or product categories with direct MTT interference effects and compiled them into an accessible reference list. We further discuss the mitigation strategies and alternative assays evaluated and/or incorporated into the validated NAM-based guidelines to improve the reliability of the data analysis and interpretation. As a case study, we discuss about an established mitigation strategy incorporated in validated NAMs that address ocular and dermal safety endpoints and based on human reconstructed tissue models. This manuscript provides a practical collection of information to support study design, identification of potential MTT-interfering compounds, and selection of appropriate mitigation strategies when MTT is used as a viability endpoint in NAM-based safety assessments.

## Highlights

• Compounds interfering with MTT assay can compromise the interpretation of viability data generated by New Approach Methodologies (NAMs).
• This article compiles and discusses compounds reported to directly interfere with the MTT endpoint.
• Practical mitigation strategies and alternative viability assays are summarized to support reliable study design and data interpretation.
• The compiled references resource supports the identification and management of potential MTT interference in NAM-based safety assessments.

## 1 Overview

New Approach Methodologies (NAMs) encompass a broad range of non-animal approaches based on *in chemico, in vitro* and *ex vivo* test systems of which several have been validated for regulatory purposes. Other modern technologies continuously enter the space of NAMs, such as organ-on-a-chip systems, organoid models, computational modeling, artificial intelligence-driven predictions, and high-throughput screening technologies. These methodologies - some originated in academic, others in industry labs - are increasingly supported toward validation and adoption by regulatory agencies to improve the human relevance of safety assessments while reducing reliance on animal testing (FDA, 2025.; FDA-NIH, 2025.; NIH, 2025a., NIH, 2025b.; European Commission, 2026.; NIH, 2026.). Besides their primary use for safety evaluations, NAMs also offer the potential to provide mechanistic insights into biological responses based on relevant, human-specific data, and to enable more efficient and reliable evaluation of large numbers of substances and formulations.

Within the NAMs framework, the assay based on the vital dye 3-(4,5-dimethyl-2- thiazolyl)-2,5-diphenyl-2H-tetrazolium bromide (MTT) remains one of the most widely used methods for evaluating cellular viability and metabolic activity. When used in conjunction with reconstructed human tissue models and other advanced *in vitro* test systems, the MTT assay serves as a key endpoint for assessing cytotoxicity, irritation potential, and tissue damage following exposure to discrete chemicals, complex mixtures, pharmaceuticals, pesticides, cosmetics and consumer products, etc. The assay is incorporated into several internationally recognized test guidelines and is frequently used to support hazard identification (OECD, 2024, 2025a, 2025b; OECD, 2025c).

An important consideration when using the MTT assay in NAM-based safety assessments is the potential for evaluated test substances themselves to interfere directly with the assay endpoint, in the absence of metabolically active biological systems (cells, tissues) able to convert MTT to its purple reduced form that can be read spectrophotometrically. Thus, this artifact leads to artificially elevated viability measurements which impact the accuracy of the data analysis and interpretation (Stepanenko and Dmitrenko, 2015). Such interactions can compromise the reliability of the assays used for safety assessments and may ultimately affect the prediction and hazard classification decisions. Consequently, many regulatory test guidelines incorporating the MTT assay require the evaluation of potential direct MTT reduction interference before interpreting viability data (OECD, 2024, 2025a, 2025b; OECD, 2025c). Appropriate controls and correction procedures are therefore essential to ensure that observed responses accurately reflect biological effects rather than chemical artifacts, thereby improving the robustness and confidence of NAM-derived safety assessments.

To support these efforts, we initiated a search to identify all available peer-reviewed manuscripts that reported data on chemical classes and product categories found to interfere directly with the MTT endpoint. In providing a comprehensive list of such materials, we aim to bring broader awareness of this challenge to the scientific community, to provide examples of mitigation strategies to circumvent this artifact, and ultimately to bolster confidence in the interpretation of MTT data, especially when used for decision making within the regulatory registration process.

## 2 MTT assay: principle, applications, challenges and limitations

The MTT assay was developed by Mosmann (Mosmann, 1983) and is based on the reduction of the yellow, water-soluble MTT to a purple, water-insoluble formazan product (Carmichael et al., 1987). Reduction of the tetrazole ring occurs through electron transfer reactions mediated by intracellular oxidoreductases and reducing equivalents such as the reduced forms of Nicotinamide Adenine Dinucleotide (NADH) and Nicotinamide Adenine Dinucleotide Phosphate (NADPH) (Berridge et al., 2005) (Fig. 1). Following solubilization, the resulting formazan can be quantified spectrophotometrically, providing a convenient colorimetric readout of cellular reducing activity (Collier and Pristos, 2003).

**Fig. 1:**
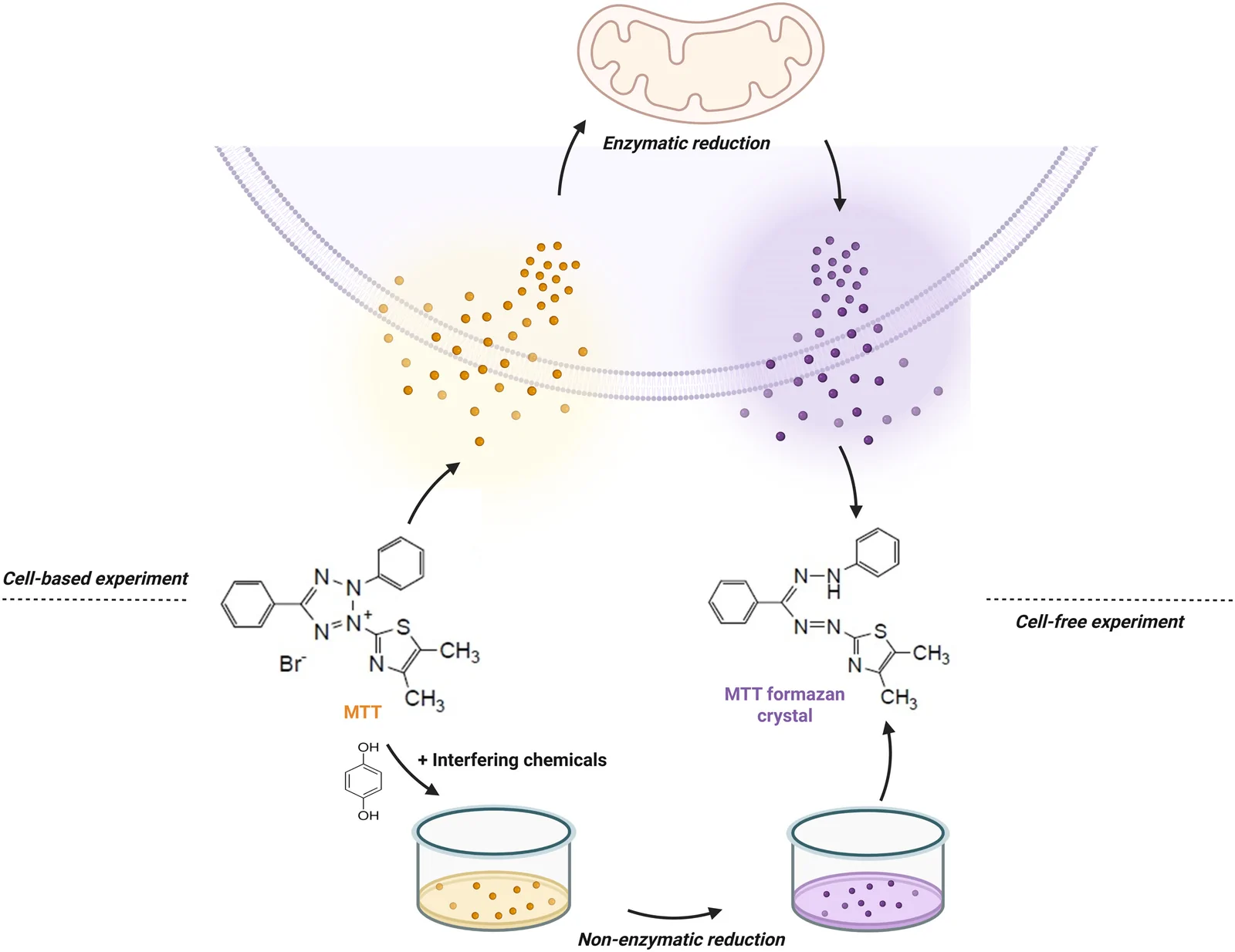
Principles of the reduction of yellow MTT to purple formazan salt by: intracellular enzymes (top panel indicated as “cell-based experiment”) or by interfering chemicals (test substances or finished products), in the absence of a metabolically active biological system (bottom panel indicated by “cell-free experiment”). Figure created in BioRender. Costin, E. (2026) https://BioRender.com/8av4wlm. Abbreviations: MTT, [3-(4,5-dimethyl-2- thiazolyl)-2,5-diphenyl-2H-tetrazolium bromide].

Because the amount of formazan generated generally correlates with the number and metabolic activity of viable cells, the MTT assay has been widely used to assess cell viability, proliferation, and cytotoxicity following exposure to chemicals, raw ingredients, pharmaceuticals, and finished products (Chacon et al., 1997; Gabrielson, 2002; Tunney et al., 2004; Wang et al., 2010; Tran, 2024). Its relatively low cost, short execution time, ease of quantification, and compatibility with high-throughput formats have contributed to its broad adoption across laboratories (Collier and Pristos, 2003; Lim et al., 2015).

However, MTT reduction reflects cellular metabolic activity rather than viable cell number directly, and metabolically active senescent or otherwise compromised cells may continue to reduce MTT (Careño et al., 2021). Therefore, the MTT assay is susceptible to both chemical and biological interference. Certain test substances can directly reduce MTT in the absence of metabolically active cells, potentially resulting in an artificial increase in measured formazan and an overestimation of viability (Fig. 1). Reported examples include ascorbic acid, resveratrol, quercetin, luteolin, and kaempferol (Chakrabarti et al., 2000; Bruggisser et al., 2002; Peng et al., 2005). Test substances may also alter cellular oxidoreductase activity and thereby increase or decrease MTT reduction independently of actual cell survival (Liu et al., 1997; Weyermann et al., 2005; Jaszczyszyn and Gąsiorowski, 2008; Ulukaya et al., 2008).

The MTT assay has been extensively researched and reported on in literature since its inception in 1983 (Mosmann, 1983). The scientific community recognized that test substances evaluated using the MTT assay may interfere with its performance and accuracy through multiple mechanisms that ultimately may impact the interpretation of data obtained. Summarized in Fig. 2 are all reported interference mechanisms of which we will focus in this manuscript strictly on the direct chemical reduction of MTT (panel A) for reasons further explained. Thus, we will only briefly summarize other points of interference as they have been the subject of extensive research captured in review manuscripts, book chapters and papers investigating specific chemical classes.

**Fig. 2:**
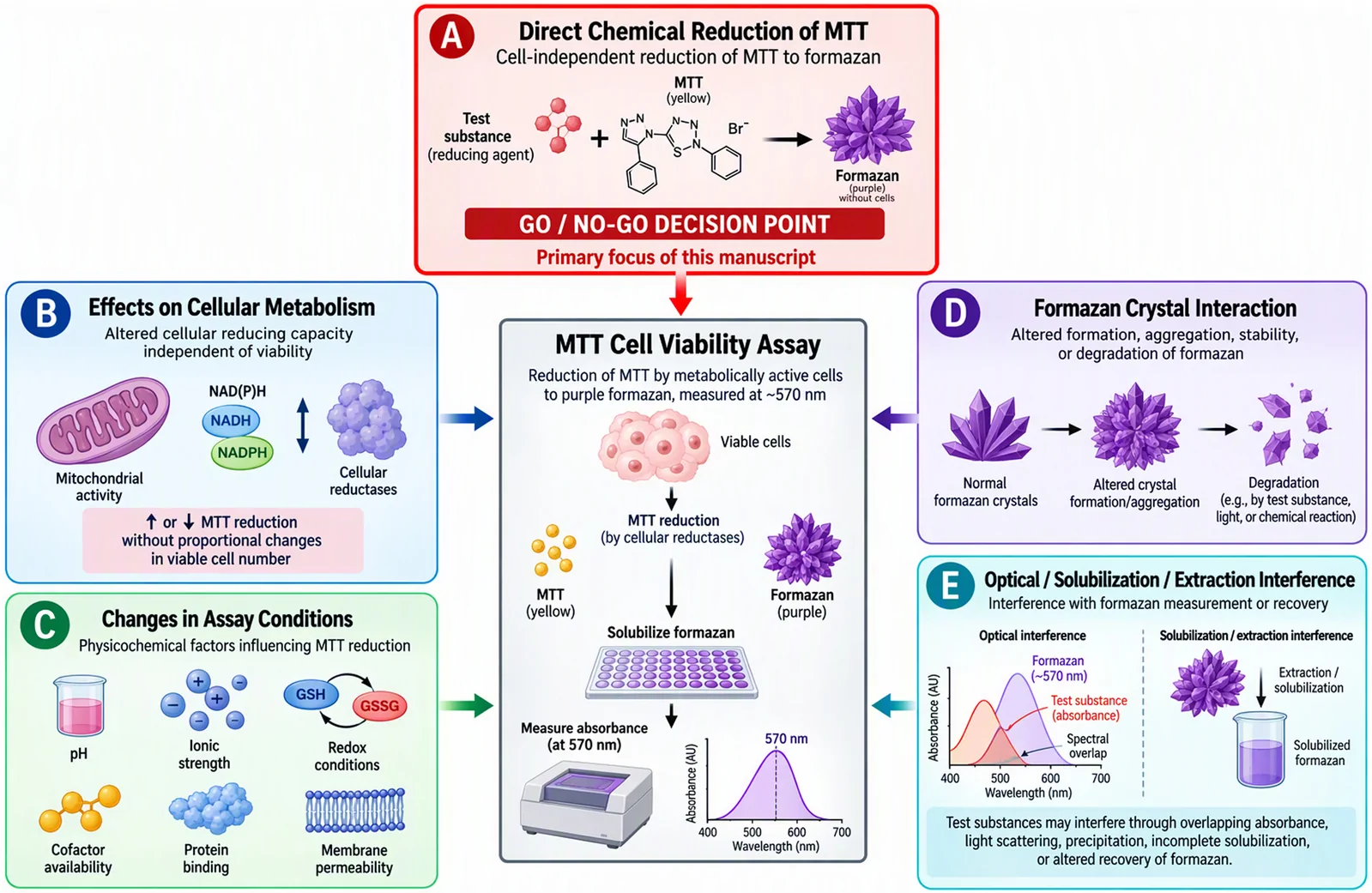
Mechanisms of test substance interference with the MTT cell viability assay. This figure summarizes the major mechanisms by which test substances may interfere with MTT-based cell viability measurements, resulting in erroneous estimation of cellular viability or cytotoxicity. The central MTT assay layout briefly explains the reduction of MTT by metabolically active cells to insoluble purple formazan, followed by solubilization and spectrophotometric measurement at approximately 570 nm. (**A**) Direct chemical reduction of MTT. Test substances may chemically convert MTT to formazan in the absence of cells, resulting in a cell-independent signal. This mechanism represents a critical binary, go/no-go decision point because direct MTT reducers can invalidate interpretation of subsequent viability measurements. (**B**) Effects of test substances on cellular metabolism. Test substances may alter mitochondrial activity, cellular reductase activity, or intracellular NAD(P)H availability without proportionally affecting viable cell number. Consequently, MTT reduction may increase or decrease independently of actual cytotoxicity. (**C**) Changes in assay conditions. Test substances (or their residues) may impact the physicochemical properties of the test system, including pH, ionic strength, redox conditions, cofactor availability, protein binding, and membrane permeability, which may subsequently influence cellular MTT reduction and contribute to changes in assay performance and results interpretation. (**D**) Formazan crystal interaction. Test substances may affect the formation, aggregation, stability, or degradation of formazan crystals, thereby altering the amount of detectable reaction product despite unchanged cellular viability. (**E**) Optical/solubilization/extraction interference. Test substances may interfere with quantification of formazan through spectral overlap, absorbance, light scattering, precipitation, incomplete formazan solubilization, or altered extraction efficiency, leading to inaccurate absorbance measurements. Figure created with 365 Microsoft Copilot. Abbreviations: MTT, [3-(4,5-dimethyl-2- thiazolyl)-2,5-diphenyl-2H-tetrazolium bromide]; NADH, Nicotinamide Adenine Dinucleotide; NADPH, Nicotinamide Adenine Dinucleotide Phosphate.

As MTT measures metabolic reducing activity, test substances may alter the NAD(P)H production, mitochondrial activity, or oxidoreductase activity without changing the cell viability *per se*. As a result, a change in the MTT signal may not be proportional to the actual number of viable cells in that reduced MTT signal does not necessarily indicate cell death and increased MTT signal may not be necessarily indicative of increased cell number (Mosmann, 1983; Berridge et al., 2005; Ghasemi et al., 2021) (Fig. 2, panel B). This distinction becomes particularly important when interpreting concentration-response relationships. Test substances can also change the conditions required for the normal MTT reaction chemistry or cellular metabolism, including variations in pH, ionic strength, membrane permeability, protein binding, availability of cofactors, oxygen/redox conditions (Mosmann, 1983; Berridge et al., 2005; Ghasemi et al., 2021; Choi et al., 2022) (Fig. 2, panel C). This suite of effects can indirectly change the measured MTT signal and thus impact the data analysis and interpretation.

Another point of test substances’ interaction with MTT is after the formazan is formed. Test substances may chemically alter the formazan, reduce or oxidize it, change its solubility or affect its extraction from cells/tissues (Stockert et al., 2012, 2018; Ghasemi et al., 2021; Choi et al., 2022) (Fig. 2, panel D). As a result, the amount of formazan measured spectrophotometrically may not accurately represent the amount generated by cellular metabolism.

Some test substances may affect the dissolution of formazan crystals or alter the efficiency of its extraction in the solvents of choice (Berridge et al., 2005; Stockert et al., 2012; Ghasemi et al., 2021) (Fig. 2, panel E). Furthermore, test substances that are intrinsically colored or that may become colored during the exposure may absorb at or near the wavelength of formazan and thus cause false absorbance readings. They can interfere simply because they have optical properties that overlap with the MTT endpoint measurement (Wang et al., 2010; Ghasemi et al., 2021; Choi et al., 2022) and thus may induce either a false increase or false decrease in the apparent MTT signal (Fig. 2, panel E). This artifact is of critical relevance when testing finished personal care or cosmetic products if they are containing pigments, dyes, botanical extracts or other optically active ingredients.

The practical characteristics and advantages of the MTT assay have also supported the extensive use of MTT as a viability endpoint in NAMs, particularly in cell- and reconstructed human tissue-based models. In validated dermal and ocular safety methods, MTT-derived tissue viability is commonly used to quantify responses following exposure to test substances/finished products and to support hazard identification, classification, or comparative assessment. Organisation for Economic Co-operation and Development (OECD) Test Guidelines have also recognized that MTT-based viability measurements may be affected by interference with conventional colorimetric readouts, particularly when test substances are intrinsically colored or absorb within the same spectral range as MTT formazan. Thus, the preliminary testing of compounds for their ability to independently induce the reduction of MTT in the absence of a metabolically active biological system is absolutely necessary and required in these assays (OECD, 2024, 2025a, 2025b; OECD, 2025c). Recognizing the importance of addressing these challenges, we discuss in our manuscript the direct chemical reduction of MTT by test substances in the absence of a metabolically active biological system. This is the first and most important interference point that is included in all commercially conducted assays, especially for regulatory purposes and that represents the go/no-go signal for the subsequent use of the MTT assay. The rest of the artifacts and interference points become obsolete if the assay should not be considered reliable to begin with. Thus, our manuscript promotes the direct MTT reduction test as the decision factor for the selection of this assay in the investigation of test substances’ impact on cellular viability (Fig. 2, panel A).

## 3 Chemical classes and product categories found to interfere with the MTT assay in the absence of a biological test system

### 3.1 Inclusion and exclusion criteria used for the generation of the database

Our database of analyzed references was constructed based on the following inclusion criteria: only publicly available, peer-reviewed sources were considered. Furthermore, the manuscripts analyzed had to report on cell-free experiments addressing any interference of test substances or finished products with the MTT endpoint. If the manuscripts also reported experiments based on biological test systems using the MTT assay, they were included in our analysis. Manuscripts that identified the interference with the MTT assays solely based on cell- or tissue-based experiments were not considered for inclusion in the database, as they were reviewed by Stepanenko and Dmitrenko (2015) and are not the primary focus of this manuscript. Such interferences may be induced by alteration of the cellular redox state or by MTT’s cellular transport rather than from direct chemical reduction of MTT. For example, dicoumarol has been reported to modify MTT’s reduction through changes in cellular redox metabolism (Collier and Pritsos, 2003), while amyloid-β was reported to induce false neuronal cytotoxicity by enhancing cellular neuronal efflux of MTT-derived material (Kerokoski et al., 2001).

The analysis of the references that met the inclusion criteria are included in Supplementary Table 1; for clarification, all cell-free experiments that investigated the interference of chemicals with the MTT assay will be referred to from hereon as “direct MTT reduction tests”. A summary of the approaches evaluating the interference of chemicals with the MTT assay in the manuscripts evaluated is provided in Supplementary Table 2. Also, for convenience the chemicals or products identified in these resources to directly reduce MTT (in the absence of a biological system) were extracted and listed in alphabetical order in Table 1, alongside their respective references.

**Table 1:** Comprehensive list of test substances or finished products reported to be direct MTT reducers (referred to as “MTT-Interfering Compounds”).

MTT-Interfering Compounds	Source	MTT-Interfering Compounds	Source
2-deoxyglucose	Van Tonder et al., 2015	Mercaptosuccinic acid	Neufeld et al., 2018
2-Ethylhexylthioglycolate	Abo et al., 2023	Methotrexate	Ulukaya et al., 2004
2,4-Pentanediol	Abo et al., 2023	Monoethanolamine (10% in aqueous solution)	Abo et al., 2023
[3-(2-Aminoethylamino)propyl]trimethoxysilane	Abo et al., 2023	m-Phenylene diamine (10% in aqueous solution)	Abo et al., 2023
3-bromopyruvate	Van Tonder et al., 2015	Myricetin	Han et al., 2010
β-mercaptoethanol	Natarajan et al., 2000	N-acetylcysteine	Bruggisser et al., 2002
*Artemisia argyi* extract	Shoemaker et al., 2004		Natarajan et al., 2000
Baicalein	Han et al., 2010	N-acetyl-d-penicillamine	Neufeld et al., 2018
bis-(3-Aminopropyl)-tetramethyldisiloxane	Abo et al., 2023	n-Butanal	Abo et al., 2023
Caffeic acid	Han et al., 2010	n-Hexanol	Abo et al., 2023
Calcium thioglycolate (10% in aqueous solution)	Abo et al., 2023	n-Octylamine	Abo et al., 2023
Catechin	Han et al., 2010	Oxidized soot	Holder et al., 2012
Chlorogenic acid	Han et al., 2010	Paclitaxel	Ulukaya et al., 2004
Cholesterol	Ahmad et al., 2006	Pamidronate	Ulukaya et al., 2004
*Cimicifuga racemosa* L. extract	Bruggisser et al., 2002	p-coumaric acid	Han et al., 2010
Cisplatin	Ulukaya et al., 2004	Polyethylene glycol-coated gold particles	Ghasemi et al., 2021
Corn oil	IIVS	Potassium hydroxide	IIVS
Cysteine	Neufeld et al., 2018	Pyrrolidine-dithiocarbamate	Natarajan et al., 2000
	Tran et al., 2024	Quercetin	Han et al., 2010
Dicaprylyl ether	Abo et al., 2023		Peng et al., 2005
Diesel particles	Holder et al., 2012		Somayaji and Shastry, 2021
Diethylethanolamine	Abo et al., 2023		Talorete et al., 2006
Diethylethanolamine (50% in aqueous solution)	Abo et al., 2023	Resveratrol	Bruggisser et al., 2002
Diethylethanolamine (25% in aqueous solution)	Abo et al., 2023		Somayaji and Shastry, 2021
Diethylethanolamine (10% in aqueous solution)	Abo et al., 2023	*Rheum palmatum*	Shoemaker et al., 2004
Di-isopropanolamine	Abo et al., 2023	Rutin	Somayaji and Shastry, 2021
Dithiothreitol	Natarajan et al., 2000		Talorete et al., 2006
	Shoemaker et al., 2004	*Salvia chinensis*	Shoemaker et al., 2004
Docetaxel	Ulukaya et al., 2004	Scutellarin	Han et al., 2010
D-Penicilliamine	Neufeld et al., 2018	Sesame oil	IIVS
*Duchesnea indica*	Shoemaker et al., 2004	Silver nanoparticles	Mello et al., 2020
Epicatechin	Han et al., 2010	Sodium hydroxide (1% in aqueous solution)	Abo et al., 2023
Epicatechin gallate	Han et al., 2010	Sodium hydroxide	IIVS
Epigallocatechin	Han et al., 2010	Soot	Holder et al., 2012
Epigallocatechin gallate	Han et al., 2010	Tetraethylene glycol diacrylate	Abo et al., 2023
	Somayaji and Shastry, 2021	Thiodiglycol	Abo et al., 2023
Epirubicine	Ulukaya et al., 2004	Titanium dioxide	Holder et al., 2012
Gallic acid	Han et al., 2010	Titanium nanoparticles	Lapu and Popescu, 2013
Gallocatechin gallate	Han et al., 2010	Triethanolamine	Abo et al., 2023
gamma-Aminopropyl triethoxy silane	Abo et al., 2023	Triethanolamine (10% in aqueous solution)	Abo et al., 2023
gamma-Mercaptopropyl trimethoxy silane	Abo et al., 2023	Triisooctylamine	Abo et al., 2023
*Gleditsia sinensis*	Shoemaker et al., 2004	*Uncaria rhynchophylla*	Shoemaker et al., 2004
Glutathione	Neufeld et al., 2018	Vitamin A	IIVS
	Tran et al., 2024	Vitamin C (Ascorbic acid)	Bruggisser et al., 2002
Glycidyl methacrylate	Abo et al., 2023		Chakrabarti et al., 2000
Graphene	Jiao et al., 2015		IIVS
*Hypericum perforatum* L. extract	Bruggisser et al., 2002		Natarajan et al., 2000
Isopropyl acetoacetate	Abo et al., 2023		Tran et al., 2024
Kaempferol	Bruggisser et al., 2002	Vitamin E	Bruggisser et al., 2002
	Han et al., 2010		IIVS
*Ligustrum lucidum*	Shoemaker et al., 2004		Natarajan et al., 2000
Lonidamine	Van Tonder et al., 2015	Vitamin E isomers (α-, β-, λ-, δ-tocotrienols and α-tocopherol)	Lim et al., 2015
Luteolin	Peng et al., 2005		
	Talorete et al., 2006		

Abbreviations: MTT, 3-(4,5-dimethyl-2- thiazolyl)-2,5-diphenyl-2H-tetrazolium bromide

Note: the MTT-interfering compounds are listed in alphabetical order.

### 3.2 Interference with the MTT assay: nanomaterials

Nanomaterials represent a category of high interest to the industry given their wide spread use across multiple product classes. Several manuscripts reported about the interference of nanoparticles of various types and sizes with the MTT assay: Holder et al., 2012; Lupu and Popescu, 2013; Jiao et al., 2015; Mello et al., 2020; Ghasemi et al., 2021 (Supplementary Table 1). Of these resources, the results reported by Mello et al. (2020) identified mechanistic reasons for the interference, specifically related to silver nanoparticles. The authors suggest that the reduction of MTT by the silver nanoparticles (AgNPs) was most likely catalyzed by silver, as AgNO_3_ was also capable of reducing MTT in a dose-dependent manner. Indeed, redox-active metals can catalyze the reduction of tetrazolium salts (Bahadar et al., 2016), and AgNPs are capable of catalyzing redox reactions (Mallick et al., 2006). Mello et al. (2020) also found that size and coating altered the magnitude of the reaction kinetics, indicating that different AgNPs can cause distinct levels of interference.

### 3.3 Interference with the MTT assay: botanicals

Components of botanicals and especially phytochemicals were reported to induce changes in the cellular metabolic activity (Plumb et al., 1989; Hsu et al., 2003). The MTT itself was found to be able to interact with some phytochemicals, resulting in false results in cell viability (Hsu et al., 2003; Ulukaya et al., 2004; Devika et al., 2008).

Multiple references captured in our database (Supplementary Table 1) reported on the impact that botanicals have on the MTT assay in the absence of a biological system: Bruggisser et al., 2002; Shoemaker et al., 2004; Peng et al., 2005; Talorete et al., 2006; Han et al., 2010; Somayaji and Shastry, 2021. For example, Peng et al., 2005 indicated that flavonoids (luteolin, quercetin) as components of botanicals are capable of reducing MTT directly. Furthermore, Somayaji and Shastry, 2021 examined the effects of bio-flavonoids and discovered instant formation of the formazan salt in the absence of cells in the MTT assay. This observation was supported by Shoemaker et al., 2004 whose results detailed significant MTT reduction by herbal extracts in the absence of living cells.

### 3.4 Interference with the MTT assay: antioxidants

Multiple reports included in our database (Supplementary Table 1) indicated that antioxidants directly interfere with the MTT assay: Chakrabarti et al., 2000; Natarajan et al., 2000; Bruggisser et al., 2002; Lim et al., 2015; Tran et al., 2024. For example, Bruggisser et al. (2002) confirmed that ascorbic acid, vitamin E, and N-acetylcysteine, components usually added to cell culture medium as cytoprotective agents, can reduce the MTT in the absence of metabolically active cell populations. The data reported by these manuscripts confirmed results previously published by Natarajan et al., 2000. Mechanistically, the reductive potential of ascorbic acid is due to its enediol group. In comparison, the phosphorylated derivative of ascorbic acid displayed no interference with the MTT assay because the enediol group was linked to a phosphate group. In analogy, vitamin E was active whereas the ester-bound vitamin E succinate was inactive.

Chakrabarti et al., 2000 also confirmed ascorbic acid as a direct MTT reducer. Interestingly, the novelty of the authors’ findings is that retinol acted as a reductase in catalyzing this reaction specifically. This is the first report showing that a nonprotein, non-RNA biomolecule acts as an enzyme.

### 3.5 Interference with the MTT assay: structural moieties

Our analysis indicated that several structural groups play specific roles in the direct reduction of MTT by a series of chemical classes. For example, thiols were identified as one major class of moieties that interfere with the MTT endpoint (Shoemaker et al., 2004; Neufeld et al., 2018). Tran et al., 2024 studied the auto-reduction of media components that participate in spontaneous redox reactions with MTT and identified conditions under which acellular formation of formazan dye occurs. One interesting finding was that the oxidation pathway of thiols does not proceed through sulfoxide or sulfone oxidation, but rather through oxidation to disulfides. In this study, ascorbic acid, cysteine, and glutathione were primarily reactive with MTT under basic conditions. As another category or structural moiety of interest, Neufeld et al., 2018 investigated the carboxyl groups and demonstrated that they are responsible for the direct reduction of MTT.

Hydroxyl groups were shown to impact the results obtained when using the MTT assay (Ahmad et al., 2006); specifically, due to its free hydroxyl groups, cholesterol enhanced exocytosis of formazan granules, causing a decrease in absorbance levels in a cell-based test system (Supplementary Table 1). Within cells, the cholesterol treatment led to certain metabolic changes which resulted in decreased formazan formation (Ahmad et al., 2006). Multiple reports pointed towards phenolic hydroxyl groups that interfered with the MTT assay (Han et al., 2010; Somayaji and Shastry, 2021). To detail, Han et al., 2010 showed that when three or more phenolic hydroxyl groups are present on a conjugated polyphenol, a significant increase in MTT reduction was observed. Furthermore, Somayaji and Shastry, 2021 showed that the phenolic group can accept an electron from fairly stable phenolic radicals. They contain a highly reactive hydroxyl group, especially on a B ring with favorable electron-donating properties. The hydroxyl group has the ability to transfer the electrons to MTT and reduces it to formazan. Flavonoids have a wide number of polyphenols that occur naturally; they vary considerably regarding the type of functional group and types of heterocycle involved and were indeed shown to be direct MTT reducers when botanical extracts were evaluated (see Section 3.3.).

Funk et al., 2007 reported that the reduction of MTT by albumin is due to its free cysteine residue, which was also observed when cysteine and glutathione were used as individual chemicals. These results might explain the observations made previously by Zhang and Cox, 1996 showing that serum used for cell culture in concentrations higher than 5% may modulate the activity of enzymes responsible for the reduction of MTT. In contrast however, Huang et al., 2004 reported that albumin decreased the signals elicited by ascorbic acid, which is known to reduce tetrazolium salts to formazans (Chakrabarti et al., 2000). Upon addition of albumin, ascorbic acid is bound to it as previously reported (Peters, 1996) and its reductive activity is inhibited.

This example of contradictory results obtained for the same compound point towards the need to evaluate any interference that may occur in the MTT-based viability assays in the specific experimental conditions rather than extrapolating from previous reports that may have generated data using slightly different designs.

### 3.6 Interference with the MTT assay: culture media components

Culture media represent an important experimental matrix that can influence MTT measurements and contribute to variability in assay performance (Shoemaker et al., 2004; Neufeld et al., 2018). As discussed in Section 3.5, several individual media constituents, including antioxidants, thiol-containing compounds, vitamins, and other redox-active molecules, are capable of interacting directly with MTT. Rather than repeating the effects of these individual chemicals, it is important to consider that their combined presence within the composition of culture medium can modify both baseline MTT reduction and the apparent activity of the test substance.

Not only is it possible for culture media components to directly reduce MTT, but they may also influence MTT reduction mediated by tested chemicals such as plant extracts (Karakaş et al., 2017), flavonoids (Peng et al., 2005; Talorete et al., 2006) or drugs (Bilmin et al., 2013). In these cases, the interference has been attributed to the presence of typical culture media nutrients/additives such as glucose, vitamins, cysteine, enzymes, etc. (Peng et al., 2005; Talorete et al., 2006).

Fetal Bovine Serum (FBS) is particularly relevant because of its complex and variable composition, which includes proteins, hormones, growth factors, lipids, cytokines, and extracellular vesicles. In addition to potential effects on cellular phenotype and metabolism, serum proteins such as albumin can bind test chemicals and thereby alter their free concentration and effective exposure to cells or tissues (Hayden et al., 2015). Batch-to-batch and supplier-related variation in FBS may therefore introduce an additional source of uncertainty in MTT-based measurements and complicate assay standardization (Jochems et al., 2002).

To reduce these uncontrolled variables, reconstructed human tissue models used in regulatory NAMs (OECD, 2024, 2025a, 2025b; OECD, 2025c) are commonly maintained in specialized serum-free or chemically defined media developed to support consistent tissue morphology, differentiation, barrier function, and assay performance. This approach improves reproducibility across laboratories and over time while minimizing matrix-related effects that could confound viability measurements (Gottipamula et al., 2013).

The broader transition toward animal-component-free and chemically defined culture systems also aligns with the Replacement, Reduction and Refinement (3Rs) principles and with the development of standardized, human-relevant NAM platforms (Sheasgreen et al., 2009; Van der Valk, 2018). These considerations further support evaluation of the complete assay matrix, in addition to the test substance itself, when assessing potential MTT interference. Section 4 provides a regulatory case study of reconstructed human tissue-based methods in which preliminary assessment of direct MTT reduction is incorporated as standard procedure.

## 4 Mitigation

### 4.1 Case study: direct MTT reduction method used in NAMs based on reconstructed tissues

When conducting studies using reconstructed tissue models and based on established, validated assays, it is necessary to assess the ability of each test substance or finished product to directly reduce MTT. Generally, the exact concentrations of reagents and reaction conditions are provided in the respective guidelines or Standard Operating Procedures (SOPs) prepared by the tissue manufacturers. As an example based on tissue models provided by MatTek Corporation (Ashland, MA, USA), when the direct MTT reduction test is conducted, the appropriate dosing volume of the test substances is combined with the MTT solution and the mixture incubated in the dark at 37 ± 1 °C in a humidified atmosphere of 5 ± 1% CO_2_ in air (standard culture conditions) for 1 to 3 hours. A negative control, usually an equivalent volume of sterile, deionized water, is tested concurrently.

In this preliminary test, if the MTT solution color turns purple, the test substance/finished product is presumed to have reduced the MTT directly, without support from a metabolically active biological system. Water insoluble test substances may show direct reduction (darkening) only at the interface with the medium. In cases where the test substance is shown to reduce MTT or is dark colored and MTT reduction could not be determined, the test substance that remains bound to the tissue after rinsing could result in a false MTT reduction signal. In these cases, to demonstrate whether possible residual test substance is acting to directly reduce the MTT, a functional check is performed using freeze-killed tissues to determine whether the test substance is binding to the tissue and leading to a false MTT reduction signal. As their name indicates, the freeze-killed tissues retain minimal to no metabolic activity which is purposely reduced/lost by repeated freeze-thaw cycles. They are used to account for any observed artifacts in cases where test substances were direct reducers of the vital dye MTT and they are handled in the assay in the same fashion as for the live tissues.

If little or no MTT reduction is observed in the test substance-treated freeze-killed control tissues, the MTT reduction observed in the test substance-treated live tissue may be ascribed to the live cell populations. If there is appreciable MTT reduction in the test substance-treated freeze-killed control tissues, additional data calculations must be taken to account for the chemical reduction, or the test substance may be considered untestable in this system.

In the manuscript authored by Huang et al., 2026, over 3000 test substances were tested using the OECD TG 439 protocol and all were assessed using the direct MTT reduction test. The results indicated that over a third of them were observed to directly reduce MTT. Without such a strict control, the dataset would have been very challenging to interpret.

Fig. 3 details how the visual observations are interpreted and used for expert judgement regarding the potential of test substances or finished products to reduce the MTT directly, in the absence of a biological system. In panel a), several concentrations of potassium hydroxide (KOH) as an example of a discrete test substance were tested, ranging from 20% to 0.5% and prepared in sterile, deionized water by volume. Also evaluated was a cream (as representative of a finished formula) and the negative control (sterile, deionized water). The MTT was prepared in media without phenol red in order to make the evaluation of potential reducers with ease due to the color contrast achieved. The intensity of the direct reduction of MTT by KOH decreased visibly alongside the decrease of its concentrations. The 20% to 5% concentrations are strong reducers of MTT based on the intense purple color of the entire wells. In comparison, the reduction by the KOH diluted from 2.5% to 1.5% is visible in reduced areas of their respective wells, often as particles of dark purple color. The 1% and 0.5% concentrations of KOH are not considered direct MTT reducers based on our experiment as their colors compare with the one of the negative control incubated with the MTT.

**Fig. 3:**
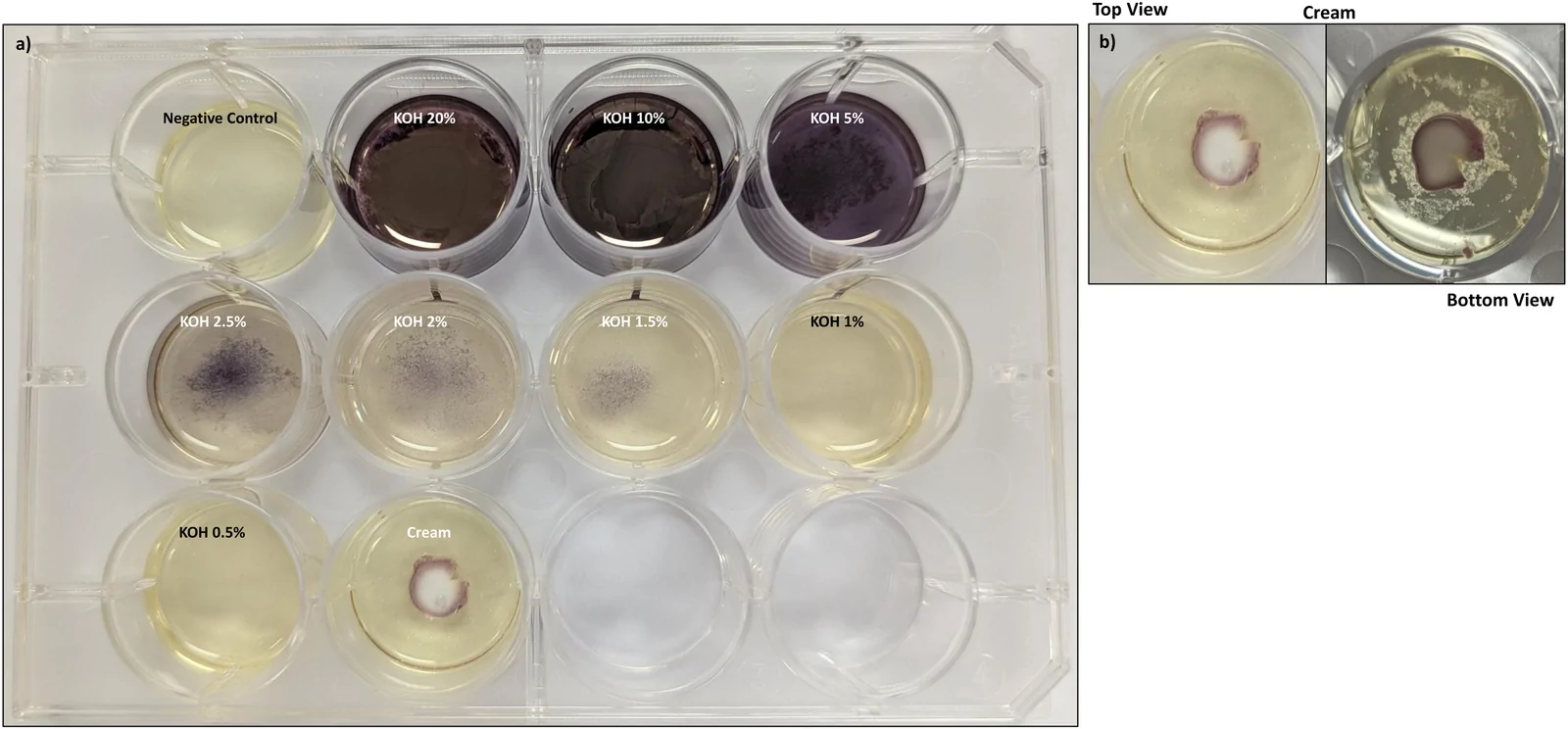
**a)** Evaluation of direct MTT reduction effects induced by test compounds such as the discrete chemical KOH or finished products (cream) alongside the negative control (sterile, deionized water). The direct MTT reduction by KOH is proportional to its concentration: all except for the 1% and 0.5% concentrations were direct MTT reducers. The cream was also determined to be a direct MTT reducer. **b)** The MTT reduction by the cream was evaluated visually from the top or bottom view of the 6-well plate for a comprehensive, reliable assessment of the interference, displayed only at the edge of the finished product. Abbreviations: KOH, potassium hydroxide; NC, negative control.

The cream included in panel a) of Fig. 3 is also considered a direct MTT reducer. The cream was evaluated from top and the bottom of the plate [Fig. 3, panel b)], where direct MTT reduction at the interface between the material and the MTT solution can be observed. The reduction along the edge is a common feature of similar finished products when evaluated in this test.

Even though the visual evaluation of the direct MTT reduction test is somewhat subjective and not based on objective measurements as all manuscripts examined and included in our database indicated, there is currently no established cut-off value set above which a chemical might be considered to interfere with MTT. The reason lies with the type of the test system used and its complexity, type of the toxicant (discrete chemical or finished product), concentration and volume of MTT established for each protocol, etc. Proper training of the personnel conducting this test is important and siding cautiously in calling a test substance or finished product a direct MTT reducer is recommended.

### 4.2 Alternative assays as mitigating strategies to MTT’s incompatibility

Alternative assays have been proposed instead of MTT as they were less or not at all impacted directly by the test substances shown to reduce the MTT. For example, assays based on: Crystal violet (Bruggisser et al., 2002), Trypan blue (Ahmad et al., 2006), Propidium iodide uptake (Ahmad et al., 2006), Adenosine Triphosphate (ATP) (Han et al., 2010), Lactate Dehydrogenase (LDH) (Holder et al., 2012), Cell Counting Kit-8 (CCK-8) (Jiao et al., 2015) or Hoechst (Mello et al., 2020) were all investigated and deemed to be less impacted by the test substances in comparison to MTT. The Neutral Red Uptake (NRU) was investigated more frequently in the manuscripts we identified and with high success over the MTT method (Lim et al., 2015; van Tonder et al., 2015; Mello et al., 2020). For this reason, the NRU assay was selected to be the endpoint of choice for the validated test method for phototoxicity (OECD, 2019). Also favored were the Resazurin (RES) assay (van Tonder et al., 2015; Neufeld et al., 2018; Mello et al., 2020) or the Sulforhodamine B (SRB) assay (van Tonder et al., 2015; Somayaji and Shastry, 2021). However, even some of these assays that were used to calibrate the performance of the MTT method have been reported to be interfered with by certain chemicals as detailed in Supplementary Table 1 [RES assay – Neufeld et al., 2018; 2,3-bis-(2‑methoxy-4-nitro-5-sulfophenyl)-2H-tetrazolium-5-carboxanilide (XTT) assay – Funk et al., 2007; Hoechst and NRU assays – Mello et al., 2020; LDH assay – Holder et al., 2012]. If any alternative assays are to be used in conjunction with established NAMs originally validated based on the MTT endpoint, proper scientific justification for the adaptation and evaluation are needed.

Alongside these alternative viability assays, we present in Supplementary Table 1 other mitigation approaches that authors reported on, such as inclusion of multiple controls, additional rinsing of interfering compounds from cells prior to conducting the MTT assay, etc. It is also worth mentioning here that for NAMs based on reconstructed tissues, the High-Performance Liquid Chromatography (HPLC) or Ultra Performance Liquid Chromatography (UPLC) methods can be used to separate and specifically quantify the MTT formazan peak for compounds that are colored or can produce overlapping optical interference. However, this method cannot inherently distinguish biologically- from chemically-produced formazan; thus, in these cases this method is better employed in combination with the freeze-killed tissue approach detailed in Section 4.1.

## 5 Summary and conclusions

The MTT assay has become a cornerstone analytical tool for generating quantitative biological data that support non-animal safety assessment strategies, owing to its robustness, simplicity, and reproducibility. One recognized shortcoming of the assay is related to the capacity of certain test substances or finished products to interfere with the mechanism of MTT reduction to its purple formazan salt and to induce the same transformation but without the contribution of a biological system that is metabolically active (Fig. 2, panel A).

We provide in our manuscript a thorough analysis of the publicly available sources (peer-reviewed manuscripts) that reported on test substances’ interference with the MTT assay. Besides identifying the test substances or finished products shown to reduce MTT directly (Table 1), in the absence of a metabolically active biological system, our analysis discussed about several alternative assays that can be used when the MTT endpoint is interfered with (Supplementary Table 1).

Several sources included in the Supplementary Table 1 agreed in concluding that preliminary testing should be conducted prior to executing experiments based on biological systems in order to allow for proper interpretation of the MTT data (Holder et al., 2012; Jiao et al., 2015; Neufeld et al., 2018; Mello et al., 2020). However, the experimental procedures described by the manuscripts we analyzed were mostly based on the reading of optical density values of the MTT - test compound mixture and comparison to controls that also vary across methods. Standardization of a direct MTT reduction test based on optical density values is challenging as it is very likely dependent on the experimental conditions such as amount/volume of the test substance, concentration and volume of the MTT, incubation conditions and length, etc. A common optical density threshold above which a compound would be considered to interfere with MTT has not been established yet and it would be relatively difficult to do so under the circumstances. Several mitigation strategies were investigated, as described in Supplementary Table 1 (Bruggisser et al., 2002; Shoemaker et al., 2004; Talorete et al., 2006; Funk et al., 2007; Holder et al., 2012; Lupu and Popescu, 2013; Jiao et al., 2015; Lim et al., 2015; Neufeld et al., 2018; Mello et al., 2020; Ghasemi et al., 2021; Somayaji and Shastry, 2021). They also vary widely across experimental setups, thus making standardization relatively difficult.

In comparison, the established method that we describe in Section 4.1 is a verified approach that was considered acceptable by the regulatory guidelines using various reconstructed tissue models. The direct MTT reduction test is based on visual observations of the test substances incubated in the presence of the vital dye and it is easy to execute. In addition, it is more conservative, as any minor change of the MTT towards a dark pink to purple color would drive the conclusion of interference with the assay and would guide the study design towards using alternative assays or the mitigation strategy based on the freeze-killed tissue controls. Unlike the subjective observations made during the conduct of the direct MTT reduction test, the use of the freeze-killed tissue controls alongside live tissues provides an objective measure of the MTT interference since it is ultimately based on optical density values read with a spectrophotometer.

The MTT assay is widely relied upon across the broad spectrum of available NAMs, particularly in validated assays based on human reconstructed tissues. To ensure reliable interpretation of the resulting data, potential interference with the assay endpoint must be carefully evaluated. The direct MTT reduction test described in Section 4.1 is an established approach for identifying and mitigating the impact of test substances that can directly reduce MTT. Importantly, this preliminary assessment has been incorporated into regulatory test guidelines, allowing testing laboratories to evaluate this potential artifact in a standardized and consistent manner. Such standardization contributes to the generation of reliable and reproducible results that can support safety assessments and regulatory registrations.

Our manuscript provides an updated and objective evaluation of the performance of the MTT assay when used to assess the toxicity of test substances and finished products in the context of safety assessments and regulatory decision-making. We also share practical knowledge, supported by data reported in the published literature and by the authors' own experimental findings, to facilitate the identification of compounds that may interfere with the MTT assay. This information can assist scientists in the design and conduct of NAM-based toxicity studies, thereby strengthening the reliability of these approaches and supporting their broader acceptance by regulatory authorities and industry. Ultimately, improving the identification and management of assay interference may help expand the application of NAMs to a wider range of safety assessment endpoints.

## Declaration of generative AI and AI-assisted technologies in the manuscript preparation process

During the preparation of this work the authors used 365 Microsoft Copilot to generate Fig. 2 and associated figure legend. After using this tool, the authors reviewed and edited the content as needed and take full responsibility for the content of the published article.

## CRediT authorship contribution statement

**Courtney Nabua:** Conceptualization. **Megan Creelman:** Data curation. **Huang Huang:** Writing – review & editing. **Nathan R. Wilt:** Methodology. **Gertrude-Emilia Costin:** Writing – original draft.

## Declaration of competing interest

The authors declare that they have no known competing financial interests or personal relationships that could have appeared to influence the work reported in this paper.

## Data Availability

No data was used for the research described in the article.

## Footnotes

Supplementary material associated with this article can be found, in the online version, at doi:10.1016/j.namjnl.2026.100148.
