# Supplementary material for: Direct Chemical Interference with the MTT viability assay: Identification and mitigation strategies in New Approach Methodologies (NAMs)
Source: NAM J. 2026 Oct 15;2:100148. doi: 10.1016/j.namjnl.2026.100148 (PMC13718833; doi:10.1016/j.namjnl.2026.100148)
Supplement: Supplementary file 1 [file mmc2.docx]

**Supplementary Table 2.** Summary of approaches evaluating interference of chemicals with the MTT assay.

| **Sources reporting on chemicals’ interference with MTT based on:** | | | |
| --- | --- | --- | --- |
| **Cell-free experiments only** | **Cell-free and cell-based experiments** | **Results of cell-based experiments using alternative assays** | **Controls included in cell-free experiments** |
| Chakrabarti et al., 2000  Funk et al., 2007  Natarajan et al., 2000  Neufeld et al., 2018  Peng et al., 2005  Shoemaker et al., 2004  Talorete et al., 2006  Tran et al., 2024  Ulukaya et al., 2004 | Abo et al., 2023  Ahmad et al., 2006  Bruggisser et al., 2002  Ghasemi et al., 2021  Han et al., 2010  Holder et al., 2012  Jiao et al., 2015  Lim et al., 2015  Lupu & Popescu, 2013  Mello et al., 2020  Somayaji & Shastry, 2021  Van Tonder et al., 2015 | Ahmad et al., 2006  Bruggisser et al., 2002  Funk et al., 2007  Han et al., 2010  Holder et al., 2012  Jiao et al., 2015  Lim et al., 2015  Mello et al., 2020  Neufeld et al., 2018  Somayaji & Shastry, 2021  Van Tonder et al., 2015 | Lupu & Popescu, 2013  Shoemaker et al., 2004  Somayaji & Shastry, 2021  Talorete et al., 2006 |

Abbreviations: MTT, [3-(4,5-dimethyl-2- thiazolyl)-2,5-diphenyl-2H-tetrazolium bromide]

Note: references are listed in alphabetical order within each column.
